# Supplementary material for: Identification of photoperiod-induced specific miRNAs in the adrenal glands of Sunite sheep (Ovis aries)
Source: Front Vet Sci. 2022 Jul 22;9:888207. doi: 10.3389/fvets.2022.888207 (PMC9354845; doi:10.3389/fvets.2022.888207)
Supplement: Supplementary file 1 [file Data_Sheet_1.ZIP › Supplementary file/miRDeep2 file.zip!/miRDeep2 file/Know_Structure/SPLP42A2/oar-mir-485.pdf]

miRBase precursor : oar-mir-485
Total read count : 1835
oar-miR-485-5p read count : 806
oar-miR-485-3p read count : 1027
remaining reads : 2

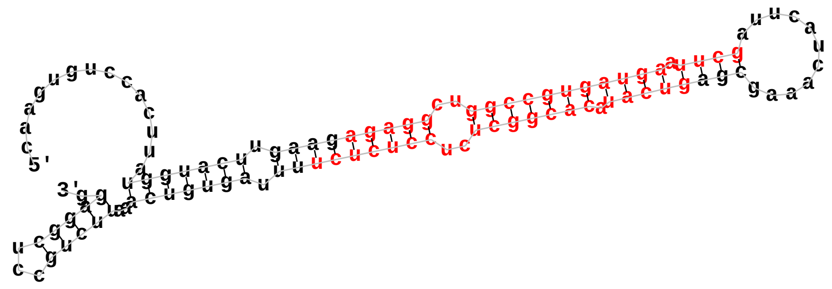

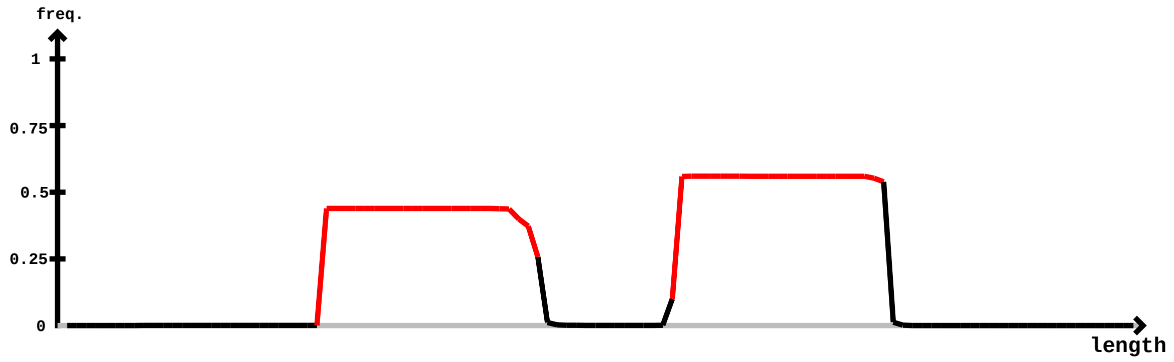


```
                                                    oar-miR-485-5p
                                                                                   oar-miR-485-3p
5'- caaguguccacuuaugguacuugaagagaggcuggccgugaugaauucgauucaucaaagcgagucauacacggcucuccucucuuuuagugucaaauucugccucggagg   -3'    exp
    ((((((.(((....)))))))))((((((((..((((((((((.((((...........)))))))).)))))))..))))))))..........(((((...))))).   reads      mm         sample
    .......ccacuuaugguacuugaag.....................................................................................   1          0          seq
    ..........................agaggcuggccgugauga...................................................................   2          0          seq
    ..........................agaggcuggccgugaugaa..................................................................   2          0          seq
    ..........................agaggcuggccgugaugaau.................................................................   68         0          seq
    ..........................agaggcuggccgugaugaauu................................................................   50         0          seq
    ..........................agaggcuggccgugaugaauuc...............................................................   212        0          seq
    ..........................agaggcuggccgugaugaauucg..............................................................   452        0          seq
    ..........................agaggcuggccgugaugaauucga.............................................................   16         0          seq
    ..........................agaggcuggccgugaugaauucgau............................................................   3          0          seq
    ..........................agaggcuggccgugaugaauucgauuc..........................................................   1          0          seq
    .................................................auucaucaaagcgagucauac.........................................   1          0          seq
    .............................................................gagucauacacggcucuccucuc...........................   1          0          seq
    ..............................................................agucauacacggcucuccucuc...........................   2          0          seq
    ..............................................................agucauacacggcucuccucucu..........................   179        0          seq
    ..............................................................agucauacacggcucuccucucuu.........................   1          0          seq
    ...............................................................gucauacacggcucuccucu............................   13         0          seq
    ...............................................................gucauacacggcucuccucuc...........................   22         0          seq
    ...............................................................gucauacacggcucuccucucu..........................   785        0          seq
    ...............................................................gucauacacggcucuccucucuu.........................   19         0          seq
    ...............................................................gucauacacggcucuccucucuuu........................   3          0          seq
    ................................................................ucauacacggcucuccucucu..........................   2          0          seq
```
